# Factors associated with depression among adolescents living with HIV in Malawi

**DOI:** 10.1186/s12888-015-0649-9

**Published:** 2015-10-26

**Authors:** Maria H. Kim, Alick C. Mazenga, Xiaoying Yu, Akash Devandra, Chi Nguyen, Saeed Ahmed, Peter N. Kazembe, Carla Sharp

**Affiliations:** 1 Baylor College of Medicine International Paediatric AIDS Initiative, Texas Children’s Hospital, Houston, USA; 2 Baylor College of Medicine Children’s Foundation Centre of Excellence Malawi, Private Bag B-397, Lilongwe 3, Malawi South Africa; 3 Department of Medicine Baylor College of Medicine, Houston, TX USA; 4 Design and Analysis Core, Baylor-UT Houston Center for AIDS Research, Houston, TX USA; 5 Department of Psychology, University of Houston, Houston, TX USA

**Keywords:** Depression, Mental health, HIV AIDS, Adolescents, Africa, Bullying

## Abstract

**Background:**

Prior research suggests that a high prevalence of depression, with a detrimental impact on treatment outcomes exists among HIV-infected youth. Data on potential risk factors of depression among HIV-infected youth in sub-Saharan Africa are scarce. This cross-sectional study aimed to identify contributory/protective factors associated with depression in Malawian adolescents 12–18 years old living with HIV.

**Methods:**

Depression was measured by a validated Chichewa version of the Beck Depression Inventory version-II (BDI-II) and the Children’s Depression Rating Scale-Revised (CDRS-R). Data on variables thought to potentially be contributory/protective were collected and included: socio-demographics, past traumatic events/stressors, behavioural factors/social support, and bio-clinical parameters. Chi-square test or two-sample *t*-test was used to explore associations between factors and depression. Additional testing via linear/logistic regression, adjusting for age and sex, identified candidate variables (p < 0.1). Final regression models included variables with significant main effects and interactions.

**Results:**

Of the 562 participants enrolled (mean age, 14.5 years [SD 2.0]; 56.1 % female), the prevalence of depression was 18.9 %. In multivariate linear regression, the variables significantly associated with higher BDI-II score were female gender, fewer years of schooling, death in the family/household, failing a school term/class, having a boyfriend/girlfriend, not disclosed or not having shared one’s HIV status with someone else, more severe immunosuppression, and bullied for taking medications. Bullying victimization was reported by 11.6 % of respondents. We found significant interactions: older participants with lower height-for-age z-scores and dissatisfied with their physical appearance had higher BDI-II scores. In multivariate logistic regression, factors significantly associated with depression were: older age, OR 1.23 (95 % CI 1.07-1.42); fewer years of schooling, OR 3.30 (95 % CI 1.54-7.05); and bullied for taking medications, (OR 4.20 (95 % CI 2.29-7.69).

**Conclusion:**

Having fewer years of schooling and being bullied for taking medications were most clearly associated with depression. Programmes to support the mental health needs of HIV-infected adolescents that address issues such as disclosure, educational support, and, most notably, bullying may improve treatment outcomes and are recommended.

## Background

An estimated 2.1 million adolescents (aged 10–19 years) are living with HIV, the vast majority in sub-Saharan Africa [1]. Nearly half of new HIV infections occur in young people (aged 15–24 years) [1].

As HIV services in sub-Saharan countries such as Malawi have successfully scaled up, improved survival rates of HIV-infected adolescents have raised awareness of the need for holistic care that takes into account mental health and quality-of-life issues. Adolescents living with HIV/AIDS are particularly vulnerable for a variety of biological, behavioural, social and structural reasons, and providing holistic care to this group presents many unique challenges [1, 2].

Globally, depression is a major contributor to the burden of disease worldwide and the number one cause of illness and disability amongst adolescents; with suicide ranking as the third most common cause of death [3]. The burden of disease is higher in low and middle-income countries [4]. Risk factors for depression in adolescents in the west include exposure to psychosocial stressors [4], bullying victimization [5–8], traumatic events, abuse [9, 10], and gender [4]. High-risk behaviours, such as earlier debut of sexual activity, substance misuse, and increased number of sexual partners, have also been associated with depression in youth [8, 11, 12]. In sub-Saharan Africa, evidence on factors potentially associated with depression amongst adolescents such as bullying [13], and substance abuse [13, 14] have just begun to emerge. Correlates, which might be particularly relevant in the African setting including orphanhood [15, 16], exposure to violence [17], poverty, and food insecurity, peer social support, and death in the family, and have been insufficiently described.

There is evidence of significant co-morbidity in patients with HIV/AIDS [18–20]. Among people living with HIV/AIDS (PLWHA) in Africa, prevalence rates for depression range between 12 % and 60 % [11, 20–25]. Our recent study of depression in HIV-infected adolescents in Malawi revealed a prevalence rate of 18.9 % [26]. Despite the increased interest in this field and the publication of several studies highlighting the high levels of depression in PLWHA, there continues to be a dearth of evidence related to adolescents and very little data examining the associated risk factors and correlates of depression in the HIV-infected adolescent population, especially in the African socio-cultural context. Studies in the West have pointed to a multifactorial aetiology of depression in HIV/AIDS that includes psychological, social, and biological factors. The few studies that have been conducted in sub-Saharan Africa suggest the following risk factors for depression in PLWHA: female gender, older age, unemployment, negative life events, childhood trauma, impaired function, poor social support, poor quality of life, and low CD4 counts [27–30]. There is a paucity of information regarding other potential correlates such as stigma, HIV associated bullying, HIV disclosure status, and self-image.

The relationship between depression and HIV appears to be bidirectional, with one exacerbating the other. Depression has been shown to worsen HIV-related outcomes, including steeper declines in CD4 counts and more rapid progression to AIDS and death [20, 25, 31, 32]. Associations also have been made with suboptimal adherence to antiretroviral therapy (ART) and treatment discontinuation, with serious implications for long-term treatment efficacy [33–35].

Malawi, a land-locked country in Southern Africa, has an adult HIV prevalence of 10.8 % [36]. The national ART programme has been successfully scaled-up, and there were approximately 486,795 patients alive and on ART as of March 2014 [37]. There is an expanding national Baylor Teen-Club programme focusing on holistic care for HIV-infected adolescents. However, awareness of mental health issues continues to be limited between both the public and healthcare providers, and few diagnostic and therapeutic options exist. Furthermore, there is little to no evidence regarding depression amongst HIV-infected adolescents in Malawi. Given the paucity of research we performed exploratory testing and included variables, based on prior research on adolescent depression, that were hypothesized to potentially contribute to or be protective of depression in HIV-infected adolescents in Malawi. We evaluated a wide range of factors: socio-demographic, past traumatic events/stressors (including bullying, self-image and school performance), behavioural factors/social support (including HIV disclosure), and bio-clinical parameters. The aim was to determine which factors remained associated once considered together, with the hope that enhanced understanding of these factors might contribute to the development of improved and comprehensive holistic adolescent ART services.

## Methods

### Study design, population and setting

We conducted a cross-sectional study to assess factors associated with depression among Malawian adolescents living with HIV. A convenience sample of n = 562 youths aged 12–18 years were recruited over a period of 8 months in 2012. The study sites were the Baylor College of Medicine – Abbott Fund Children’s Clinical Centre of Excellence (COE) and Zomba Central Hospital Tisungane ART Clinic. Zomba Tisungane ART Clinic is the main referral HIV clinic in southeastern Malawi, with more than 200 adolescents receiving care. Baylor COE, located in Lilongwe, the capital of Malawi, is the largest paediatric HIV clinic in Malawi, with more than 400 children receiving care. The COE serves as a national referral centre for paediatric and adolescent HIV care, and patients come from diverse regional and socioeconomic backgrounds.

### Study procedures

Written informed assents and consents were obtained from the participants and caregivers, respectively. Upon assenting/consenting, participants completed the Chichewa version of the Beck Depression Inventory version II (BDI-II) [26], followed by a structured clinical interview using the Children’s Depression Rating Scale – Revised (CDRS-R). Interviewers were blinded to the results of the BDI-II. Finally, a socio-demographic behavioural questionnaire (SDBAQ) was administered. A mental health clinical officer trained clinician interviewers, with mental health training and over two years of experience in adolescent HIV care, in the administration of the CDRS-R [26]. Selected HIV-related clinical variables were extracted from patient electronic medical records and health passports. All participants with severe depression or suicidal ideation received counselling from onsite mental health professionals.

### Measures

Depression was measured by a Chichewa version of the BDI-II [26] and the CDRS-R. Validation of these instruments is reported fully elsewhere [26]. In brief, the BDI-II is a self-rated depression screening instrument with 21 items and assesses the presence and severity of depressive symptoms in those ≥13 years of age [38]. It has been validated in a range of settings [39–41]. The CDRS-R is a clinician interview instrument used to both diagnose depression and to measure response to treatment in children [26, 42, 43]. It is one of the most widely used paediatric depression scales in international clinical research trials [26, 42, 44–46].

Data were collected (SDBAQ and medical record data extraction) on variables thought to be potentially associated with depression. Data included:

1. *Socio-demographic factors*: gender, age, education, family income level, location of their home, distance from clinic, primary caregiver type (father/mother, both parents, uncle/aunt, other/grandparent)
2. *Past traumatic events/stressors*: maternal death or employment status, change of caregiver, number of deaths in the family, failed school term, history of sexual or domestic violence, victim of teasing/bullying for one’s appearance or for taking medications, hospital admissions in the past year
3. *Behavioural factors/social support*: involvement in a romantic relationship, satisfaction with physical appearance, alcohol and drug use, tobacco use, HIV disclosure to another person, child’s knowledge of HIV status and age at disclosure
4. *Bio-clinical parameters*: ART use, efavirenz-based regimen, second-line ART, history of TB, initial and most recent WHO stage, initial and most recent CD4/immunological classification, prior diagnosis of depression, nutritional status, BMI-for-age z-score, height-for-age z-score.

### Statistical analysis for the depression manuscript

Descriptive statistics, such as, mean, standard deviation (SD) for continuous variables, and frequency and proportion for categorical variables, were calculated among all subjects and by groups. For the BDI-II score, we performed explorative analysis using two-sample *t*-test, analysis of variance (ANOVA) or Pearson correlation. For CDRS-R score, we defined depression by having a score equal to or higher than 55 [26]. Chi-square test or two-sample *t*-test was used to explore the association between potential factors and depression. We collapsed categories for some variables with sparse cells or with similar outcome values. For example, we collapsed ‘not in school’ (n = 3) with ‘Junior primary school’, ‘tertiary’ (n = 4) with ‘junior/senior secondary school’ in meaningful way; we also combined the categories of ‘mother alone’ and ‘father alone’ for caregiver since the results demonstrated a similar degree of depression for two separate categories (19 % and 18 % in depression by CDRS respectively). For correlated variables that measured similar features, we either combined the variables or selected the most important variable for further analysis. For example, we combined three variables related to violence (forced sex, physical abuse and witness physical violence in the home) into one variable with yes indicating experience on any of three; similarly we had two variables that referred to the caregiver, ‘who currently cares for this teenager’ and ‘who do you live with’. We chose to use the former since it is more relevant/meaningful to the provision of supportive HIV care. Malnutrition was categorized according to the National Center for Health Statistics reference standards. Z-scores for BMI and height-for-age were calculated using WHO growth standards

We assessed all variables thought to be potentially correlated with depression based on previously published research. The initial univariate analysis identified 28 variables thought to be possibly associated with depression (*p*-value less than 0.25 or *p*-value less than 0.25 or associations postulated based on previously published research). We reassessed these variables by univariate linear/ logistic regression, and then we performed a second round of screening by adjusting for age and sex using linear/logistic regression (Tables 1 and 2). Eighteen candidates with *p*-values <0.1 from either model were entered into the multivariate regressions. For both the linear and logistic regressions, we included age and sex in the models, regardless of the statistical significance, and performed backwards selection on other variables with significance ≤ 0.05. Two-way interactions were checked. Only biologically meaningful and significant interactions (such as age*satisfaction with appearance, and age*height) were retained. We rescaled age (age minus 12) so the main effect term of age could be meaningfully interpreted in the model that included interactions with age. Final models were checked by model diagnostic techniques such as residual analysis and influence statistics. Final models were checked by model diagnostic techniques such as residual analysis and influence statistics. For the logistic regression, overall fit was also assessed by Hosmer and Lemeshow Goodness-of-Fit. The beta coefficient from the linear regression, the odds ratio (OR) from logistic regression, and their 95 % confidence interval (CI) were reported to evaluate the association between the variable and outcome adjusting for other covariates. A *p*-value <0.05 was considered statistically significant, and SAS software version 9.3 (SAS Institute, Cary, N.C.) was used for all analyses.

**Table 1:** Factors associated with BDI-II score (unadjusted and adjusted for sex and age)

Variable		Mean (SD, N)/*N* (%)	Unadjusted Beta Coef (95 % CI)	Unadjusted *p*-value	Adjusted Beta Coef(95 % CI)	Adjusted *p*-value
Socio-demographic factors						
School Grade						
	Not in school or Junior Primary School	176 (31.3)	2.37 [0.68-4.06]	0.02	5.25 [3.23-7.26]	<0.0001
	Senior Primary School	229 (40.7)	1.62 [0.02-3.22]		3.5 [1.78-5.22]	
	Secondary School or Tertiary	157 (27.9)	Reference		Reference	
Family’s estimated combined income						
	Less than 14,000 MK per month	171 (31.8)	0.36 [−1.29-2.02]	0.61	0.38 [−1.26-2.02]	0.64
	14,000 -49,999 MK per month	117 (21.8)	−0.85 [−2.69-0.99]		−0.79 [−2.61-1.03]	
	More than 50,000 per month	71 (13.2)	−0.48 [−2.65-1.68]		−0.37 [−2.52-1.77]	
	I do not know	178 (33.1)	Reference		Reference	
Location of home						
	In the city	369 (65.7)	−1.24 [−2.98-0.50]	0.06	−1.49 [−3.22-0.25]	0.06
	Just outside the city	93 (16.5)	0.74 [−1.48-2.97]		0.3 [−1.92-2.52]	
	Rural area	100 (17.8)	Reference		Reference	
Time it takes to get to the clinic from home						
	0-30minutes	71 (12.7)	−0.88 [−2.96-1.20]	0.71	−0.92 [−2.98-1.15]	0.67
	31-60 minutes	229 (40.8)	−0.15 [−1.56-1.25]		−0.36 [−1.76-1.04]	
	>60 minutes	261 (46.5)	Reference		Reference	
Primary caregiver type						
	Father/Mother	178 (31.7)	−1.63 [−3.39-0.13]	0.01	−1.36 [−3.12-0.39]	0.02
	Both parents	112 (19.9)	−3.39 [−5.36--1.42]		−3.09 [−5.06--1.12]	
	Uncle/Aunt	138 (24.6)	−1.5 [−3.37-0.37]		−1.32 [−3.18-0.54]	
	Other/Grandparent	134 (23.8)	Reference		Reference	
Past-traumatic events/stressors						
Maternal death or employment status						
	Not working	106 (18.9)	−0.95 [−2.71-0.80]	0.01	−0.8 [−2.55-0.95]	0.03
	Self-employed	103 (18.3)	−1.51 [−3.29-0.26]		−1.37 [−3.14-0.40]	
	Employed by someone else	73 (13)	−3.22 [−5.24--1.19]		−2.97 [−4.99--0.95]	
	Died	280 (49.8)	Reference		Reference	
Change in caregiver						
	No change in caregiver	422 (75.2)	−1.59 [−3.10--0.08]	0.04	−1.59 [−3.09--0.09]	0.04
	Caregiver has changed once or more	139 (24.8)	Reference		Reference	
Experience of family/household deaths						
	Nobody in my family has died	149 (26.5)	−2.42 [−3.89--0.95]	0.001	−2.51 [−3.97--1.05]	0.0008
	One or more people have died	413 (73.5)	Reference		Reference	
Failed school term/class						
	No	327 (58.9)	−1.64 [−2.97--0.31]	0.02	−1.73 [−3.05--0.41]	0.01
	Yes	228 (41.1)	Reference		Reference	
Experience of forced sex, physical abuse, or witnessed physical violence in the home						
	No	477 (84.9)	−3.16 [−4.97--1.35]	0.0006	−2.97 [−4.79--1.15]	0.001
	Yes	85 (15.1)	Reference		Reference	
Experience of being bullied for one’s physical appearance						
	No	427 (76.1)	−3.11 [−4.62--1.59]	0.0001	−2.78 [−4.31--1.25]	0.0004
	Yes	134 (23.9)	Reference		Reference	
Experience of being bullied for taking medicines						
	No	459 (88.4)	−5.7 [−7.75--3.64]	<0.0001	−5.3 [−7.40--3.19]	<0.0001
	Yes	60 (11.6)	Reference		Reference	
Hospital admissions in the past year						
	No	510 (90.7)	−2.08 [−4.34-0.17]	0.07	−2.13 [−4.36-0.10]	0.06
	Yes	52 (9.3)	Reference		Reference	
Behavioral factors/social support						
Experience of being in a romantic relationship that did not involve sex						
	Never had a boyfriend/girlfriend	479 (85.4)	−3.52 [−5.35--1.69]	0.0002	−2.96 [−4.96--0.96]	0.004
	Yes, in the past or current	82 (14.6)	Reference		Reference	
Satisfaction with the way I look (physical appearance)						
	I am very happy with the way I look	460 (82)	−4.65 [−6.31--2.99]	<0.0001	−4.34 [−6.00--2.68]	<0.0001
	I am somewhat/ not at all satisfied with the way I look	101 (18)	Reference		Reference	
Use of alcohol in the past 30 days						
	Never	547 (97.3)	−7.76 [−11.77--3.74]	0.0002	−7.35 [−11.35--3.36]	0.0003
	Once a month or more	15 (2.7)	Reference		Reference	
HIV disclosure status*						
	Disclosed and have shared with someone	273 (48.6)	0.19 [−1.59-1.97]	0.09	0.98 [−2.86-0.97]	0.02
	Disclosed, have not shared with anyone	184 (32.7)	1.68 [−0.21-3.57]		0.98 [−0.74-3.12]	
	Not disclosed	105 (18.7)	Reference		Reference	
Age at disclosure		12.35 (1.63, 457)	0.55 [0.10-1.00]	0.02	0.42 [−0.09-0.92]	0.11
Bio-clinical parameters						
Child on ART						
	No	36 (6.4)	0.83 [−1.84-3.50]	0.54	0.99 [−1.66-3.64]	0.46
	Yes	526 (93.6)	Reference		Reference	
Efavirenz based ART regimen						
	No	526 (93.6)	−0.71 [−3.38-1.96]	0.60	−0.71 [−3.36-1.95]	0.60
	Yes	36 (6.4)	Reference		Reference	
Second line ART regimen						
	No	491 (87.4)	0.68 [−1.29-2.65]	0.50	0.65 [−1.33-2.63]	0.52
	Yes	71 (12.6)	Reference		Reference	
History of TB treatment						
	No	309 (55)	0.77 [−0.54-2.09]	0.25	0.75 [−0.55-2.06]	0.26
	Yes	253 (45)	Reference		Reference	
Initial WHO stage						
	WHO stage 1-2	160 (28.7)	−0.12 [−1.57-1.34]	0.87	0.03 [−1.42-1.47]	0.97
	WHO Stage 3-4	398 (71.3)	Reference		Reference	
Most recent CD4 count		516 (307, 509)	0 [−0.01--0.00]	0.009	0 [−0.00--0.00]	0.02
HIV immunological classification (based on CD4)						
	None or not significant	235 (46.2)	−2.34 [−4.18--0.49]	0.03	−2.26 [−4.09--0.43]	0.05
	Mild	112 (22)	−0.94 [−3.05-1.18]		−1 [−3.09-1.09]	
	Advanced	68 (13.4)	−0.16 [−2.56-2.25]		−0.15 [−2.53-2.22]	
	Severe	94 (18.5)	Reference		Reference	
Current nutritional status						
	Normal	544 (96.8)	−2.44 [−6.15-1.28]	0.20	−3.14 [−6.83-0.56]	0.10
	Malnourished	18 (3.2)	Reference		Reference	
BMI for age z-score		−0.88 (1.18, 561)	0.08 [−0.48-0.63]	0.79	−0.19 [−0.76-0.39]	0.52
Height for age z-score		−1.79 (1.16, 561)	−0.98 [−1.54-0.42]	0.0006	−1.31 [−1.88-0.74]	<0.0001

**Table 2:** Factors associated with depression (CDRS-R score >55) (unadjusted and adjusted for sex and age)

	Variable	Mean (SD, *N*)/*N* (%)	*N* (%)	Unadjusted OR (95 % CI)	Unadjusted *p*-value (overall)	Adjusted OR (95 % CI)	Adjusted *p*-value (overall)
Socio-demographic factors							
School Grade							
	Not in school/Junior Primary School	176 (31.3)	35 (19.9)	1.45 [0.81-2.57]	0.27	2.82 [1.39-5.73]	0.009
	Senior Primary School	229 (40.7)	48 (21)	1.54 [0.90-2.66]		2.36 [1.29-4.32]	
	Secondary School or Tertiary	157 (27.9)	23 (14.6)	Reference		Reference	
Family’s estimated combined income							
	Less than 14,000 MK per month	171 (31.8)	36 (21.1)	1.32 [0.77-2.25]	0.24	1.33 [0.77-2.28]	0.23
	14,000 -49,999 MK per month	117 (21.8)	16 (13.7)	0.78 [0.40-1.51]		0.79 [0.41-1.54]	
	More than 50,000 per month	71 (13.2)	17 (23.9)	1.55 [0.79-3.04]		1.61 [0.82-3.16]	
	I do not know	178 (33.1)	30 (16.9)	Reference		Reference	
Location of home							
	In the city	369 (65.7)	67 (18.2)	0.83 [0.48-1.45]	0.81	0.78 [0.45-1.36]	0.69
	Just outside the city	93 (16.5)	18 (19.4)	0.9 [0.45-1.83]		0.81 [0.40-1.65]	
	Rural area	100 (17.8)	21 (21)	Reference		Reference	
Time it take to get to the clinic from home							
	0-30minutes	71 (12.7)	14 (19.7)	1.25 [0.64-2.43]	0.38	1.22 [0.62-2.40]	0.50
	31-60 minutes	229 (40.8)	49 (21.4)	1.38 [0.88-2.17]		1.31 [0.83-2.08]	
	>60 minutes	261 (46.5)	43 (16.5)	Reference		Reference	
Primary caregiver type							
	Father/Mother	178 (31.7)	33 (18.5)	0.64 [0.38-1.10]	0.04	0.68 [0.39-1.17]	0.07
	Both parents	112 (19.9)	13 (11.6)	0.37 [0.19-0.74]		0.4 [0.20-0.81]	
	Uncle/Aunt	138 (24.6)	25 (18.1)	0.63 [0.35-1.12]		0.64 [0.36-1.15]	
	Other/Grandparent	134 (23.8)	35 (26.1)	Reference		Reference	
Past-traumatic events/stressors							
Maternal death or employment status							
	Not working	106 (18.9)	19 (17.9)	0.77 [0.43-1.36]	0.21	0.8 [0.45-1.43]	0.31
	Self-employed	103 (18.3)	14 (13.6)	0.55 [0.29-1.04]		0.58 [0.30-1.09]	
	Employed by someone else	73 (13)	11 (15.1)	0.62 [0.31-1.26]		0.67 [0.33-1.36]	
	Died	280 (49.8)	62 (22.1)	Reference		Reference	
Change in caregiver							
	No change in caregiver	422 (75.2)	70 (16.6)	0.57 [0.36-0.90]	0.02	0.57 [0.36-0.90]	0.02
	Caregiver has changed once or more	139 (24.8)	36 (25.9)	Reference		Reference	
Experience of family/household deaths							
	Nobody in my family has died	149 (26.5)	22 (14.8)	0.68 [0.41-1.13]	0.14	0.67 [0.40-1.12]	0.12
	One or more people have died	413 (73.5)	84 (20.3)	Reference		Reference	
Failed school term/class							
	No	327 (58.9)	55 (16.8)	0.78 [0.51-1.20]	0.26	0.76 [0.49-1.17]	0.21
	Yes	228 (41.1)	47 (20.6)	Reference		Reference	
Experience of forced sex, physical abuse, or witnessed physical violence in the home							
	No	477 (84.9)	79 (16.6)	0.43 [0.25-0.71]	0.001	0.45 [0.26-0.76]	0.003
	Yes	85 (15.1)	27 (31.8)	Reference		Reference	
Experience of being bullied for one’s physical appearance							
	No	427 (76.1)	70 (16.4)	0.53 [0.34-0.85]	0.008	0.58 [0.36-0.93]	0.02
	Yes	134 (23.9)	36 (26.9)	Reference		Reference	
Experience of being bullied for taking medicines							
	No	459 (88.4)	73 (15.9)	0.23 [0.13-0.41]	<0.0001	0.27 [0.15-0.48]	<0.0001
	Yes	60 (11.6)	27 (45)	Reference		Reference	
Hospital admissions in the past year							
	No	510 (90.7)	92 (18)	0.6 [0.31-1.15]	0.12	0.58 [0.30-1.13]	0.11
	Yes	52 (9.3)	14 (26.9)	Reference		Reference	
Behavioral factors/social support							
Past history of being in a romantic relationship that did not involve sex							
	Never had a boyfriend/girlfriend	479 (85.4)	83 (17.3)	0.54 [0.31-0.92]	0.02	0.65 [0.36-1.18]	0.16
	Yes, in the past or current	82 (14.6)	23 (28)	Reference		Reference	
Satisfaction with the way I look (physical appearance)							
	I am very happy with the way I look	460 (82)	77 (16.7)	0.5 [0.30-0.82]	0.006	0.54 [0.33-0.89]	0.02
	I am somewhat/ not at all satisfied with the way I look	101 (18)	29 (28.7)	Reference		Reference	
Use of alcohol in the past 30 days							
	Never	547 (97.3)	100 (18.3)	0.34 [0.12-0.96]	0.04	0.37 [0.13-1.07]	0.07
	Once a month or more	15 (2.7)	6 (40)	Reference		Reference	
HIV disclosure status*							
	Disclosed and have shared with someone	273 (48.6)	51 (18.7)	1.19 [0.65-2.17]	0.64	0.90 [0.47-1.75]	0.54
	Disclosed, have not shared with anyone	184 (32.7)	38 (20.7)	1.35 [0.72-2.53]		1.19 [0.62-2.28]	
	Not disclosed	105 (18.7)	17 (16.2)	Reference		Reference	
	Age at disclosure	12.3 (1.6, 4.6)		1.27 [1.11-1.47]	0.0008	1.31 [1.11-1.56]	0.002
Bio-clinical parameters							
	Child on ART						
	No	36 (6.4)	4 (11.1)	0.52 [0.18-1.50]	0.23	0.54 [0.18-1.56]	0.25
	Yes	526 (93.6)	102 (19.4)	Reference		Reference	
Efavirenz based ART regimen							
	No	526 (93.6)	103 (19.6)	2.68 [0.81-8.90]	0.11	2.75 [0.82-9.19]	0.10
	Yes	36 (6.4)	3 (8.3)	Reference		Reference	
Second line ART regimen							
	No	491 (87.4)	94 (19.1)	1.16 [0.60-2.25]	0.65	1.18 [0.60-2.31]	0.63
	Yes	71 (12.6)	12 (16.9)	Reference		Reference	
History of TB treatment							
	No	309 (55)	61 (19.7)	1.14 [0.74-1.74]	0.56	1.13 [0.74-1.75]	0.56
	Yes	253 (45)	45 (17.8)	Reference		Reference	
Initial WHO stage							
	WHO stage 1-2	160 (28.7)	28 (17.5)	0.88 [0.55-1.43]	0.61	0.91 [0.56-1.47]	0.71
	WHO Stage 3-4	398 (71.3)	77 (19.3)	Reference		Reference	
	Most recent CD4 count	516 (307, 509)		1 [1.00-1.00]	0.08	1 [1.00-1.00]	0.12
HIV immunological classification							
	None or not significant	235 (46.2)	32 (13.6)	0.58 [0.31-1.08]	0.04	0.6 [0.32-1.13]	0.05
	Mild	112 (22)	15 (13.4)	0.57 [0.27-1.19]		0.57 [0.27-1.19]	
	Advanced	68 (13.4)	18 (26.5)	1.33 [0.64-2.77]		1.35 [0.64-2.82]	
	Severe	94 (18.5)	20 (21.3)	Reference		Reference	
Current nutritional status							
	Normal	544 (96.8)	100 (18.4)	0.45 [0.17-1.23]	0.12	0.38 [0.13-1.05]	0.06
	Malnourished	18 (3.2)	6 (33.3)	Reference		Reference	
	BMI for age z-score	−0.88 (1.18, 561)		0.96 [0.80-1.14]	0.61	0.89 [0.74-1.08]	0.24
	Height for age z-score	−1.79 (1.16, 561)		0.93 [0.78-1.12]	0.46	0.87 [0.72-1.06]	0.17

### Ethics approvals

Ethical approvals for this study were granted by the National Health Sciences Research Committee (Malawi) and the Baylor College of Medicine Institutional Review Board (USA).

## Results

The clinics provided a list of 695 potential participants. Of these, 102 could not be contacted (death, loss-to-follow-up, insufficient contact information, and transfer-out to another facility); 11 could not participate due to intellectual disability. Of the remaining 582, 97 % (562) consented/assented and enrolled in the study. The mean age of participants was 14.5 years (SD 2.0), and 315 (56.1 %) were female.

### Univariate analysis of associations between BDI-II score and other variables

Mean BDI-II score for the total sample (*n* = 562) was 11.9 (SD 7.9) (Table 1, *p* < 0.1). After adjustment for age and sex, a higher BDI-II score was potentially associated with fewer years of schooling, living in a rural area or outside the city, primary caregiver type (other/grandparent), maternal death, change in caregiver, death in the family/household, failing a school term/class, experience of forced sex, physical abuse or witnessed physical violence in the home, bullied for one’s physical appearance or for taking medicines, hospital admission in the past year, experience of being in a romantic relationship, dissatisfaction with physical appearance, use of alcohol, being disclosed to and not sharing one’s HIV status with someone else, higher recent CD4 count/worse immunological stage, being malnourished, or lower height-for-age z-score. Bullying victimization for taking medications was reported by 11.6 % of respondents.

### Univariate analysis of associations between depression and other variables

The prevalence of depression as measured by the CDRS-R was 18.9 % (106/562) (Table 2, *p* < 0.1). After adjustment for age and sex, depression was potentially associated with fewer years of schooling, primary caregiver type (other/grandparent), change in caregiver, experience of forced sex, physical abuse or witnessed physical violence in the home, bullied for one’s physical appearance or for taking medicines, dissatisfaction with physical appearance, use of alcohol, older age at disclosure, worse immunological stage, and being malnourished.

### Multivariate analysis of associations between BDI-II score and other variables

In multivariate analysis (Table 3), the variables significantly associated with higher BDI-II score were female gender, fewer years of schooling, death in the family/household, failing a school term/class, being teased for taking medications, being in a romantic relationship, not disclosed to or being disclosed to but not having shared one’s HIV status with someone else, and worse level of immunosuppression. In addition, significant interactions were found between age and satisfaction with one’s physical appearance and between age and height-for-age z-score (HAZ). Older age was associated with increased BDI-II score, and the effect of age on BDI-II depended on satisfaction with one’s physical appearance and HAZ. Specifically, the negative values for the interaction terms imply that the more a patient was satisfied with his/her physical appearance or the higher the HAZ, the less was the effect of age on the BDI-II score. Thus, older participants with lower HAZ and who were dissatisfied with their physical appearance had higher BDI-II scores.

**Table 3:** Final Multivariate Linear Regression Model of factors associated with depression as measured by BDI-II

Variable	Beta Coefficient [95 % CI]	*P*-value
Age	0.54 [−0.38, 1.46]	0.25
Female	2.13 [0.82, 3.43]	0.002
Grade		
Not in school/Junior Primary School	3.84 [1.71, 5.98]	0.0005
Senior Primary School	3.17 [1.45, 4.89]	
Secondary School or Tertiary	Reference	
Nobody in my family has died	−1.77 [−3.15, −0.39]	0.01
Did not fail school term/class	−1.46 [−2.76, −0.17]	0.03
Bullying for taking medication	5.31 [3.19, 7.43]	<0.0001
Never had a boyfriend/girlfriend	−2.38 [−4.35, −0.41]	0.02
Satisfaction with physical appearance- happy with the way I look	−1.06 [−3.74, 1.63]	0.44
HIV disclosure status		
Disclosed and have shared with someone	−1.83 [−3.79, 0.13]	0.02
Disclosed, have not shared with anyone	0.23 [−1.65, 2.10]	
Not disclosed	Reference	
Level of immunosuppression		
None or not significant	−2.58 [−4.29, −0.87]	0.0009
Mild	−0.10 [−2.08, 1.88]	
Advanced	0.23 [−2.00, 2.47]	
Severe	Reference	
Height for age z-score	0.23 [−0.67, 1.12]	0.62
Age* satisfaction with physical appearance interaction	−0.93 [−1.74, −0.11]	0.03
Age* Height for age z-score interaction	−0.39 [−0.68, −0.11]	0.007

### Multivariate analysis of associations between depression and other variables

In logistic regression (Table 4), fewer variables were significantly associated with depression: age, fewer years of schooling, and bullied for taking medications. Those who were bullied had increase odds (OR 4.20 [95 % CI 2.29-7.69]) [25] of having depression.

**Table 4:** Final Multivariate Logistic Regression Model of factors associated with depression as measured by CDRS-R*

Variable	OR [95 % CI]	*P*-value
Age	1.23 [1.07, 1.42]	0.004
Female	1.43 [0.89, 2.29]	0.14
Grade		
Not in school/ Junior Primary School	3.30 [1.54, 7.05]	0.005
Senior Primary School	2.62 [1.37, 5.03]	
Secondary School or Tertiary	Reference	
Bullied for taking medication	4.20 [2.29, 7.69]	<0.0001

*As measured by CDRS-R

## Discussion

This is the first study to examine depression and associated factors among adolescents living with HIV in Malawi.

In multivariate linear regression, we found that a higher BDI-II score was associated with female gender, fewer years of schooling, death in the family/household, failing a school term/class, being bullied for taking medications, being in a romantic relationship, not being disclosed to or being disclosed to but not having shared one’s HIV status with someone else, and immunosuppression. Older participants with lower HAZ and dissatisfaction with their physical appearance had higher BDI-II scores.

Multivariate logistic regression with depression as the dependent variable identified much fewer associations, with only three variables significantly associated with depression in both the linear and logistic models: older age, fewer years of schooling, and bullied for taking medications. The identification of fewer associations in logistic regression is partially explained by inadequate power related to analysing categorical vs. continuous outcomes. In addition, the results may reflect differences in the construct being measured (continuous vs. categorical) or the scales themselves.

There is a paucity of studies examining factors associated with adolescent depression in sub-Saharan Africa. However, the relationship between increasing age in adolescence and depression has been documented in studies from the developed world [47], and an association with suicidality has been noted in a study of HIV-infected adolescents in Kenya [25]. Our study identified an association between schooling and depression: specifically, fewer years of schooling (in both linear/logistic regression models) and failing a school term (linear regression) increased the likelihood of depression. The aforementioned study from Kenya [25] also identified a relationship between not being in an age-appropriate class and having a psychiatric disorder. Although intelligence and cognitive deficits related to HIV infection were not assessed in the present study, other studies have found higher cognitive deficits [48–50] and overall lower IQ scores in HIV-infected children [51]. Additional associations found to be related to depression in prior studies that were also found to be related in the linear regression include female gender [39], the presence of immunosuppression [52], disclosure [53, 54], and death in the family.

As compared to prior studies in the west or sub-Saharan Africa amongst non-HIV infected adolescents, several HIV-related potential risk factors for depression were highlighted in the present study. Specifically, stunting, immunosuppression, dissatisfaction with one’s physical appearance, and fewer years of or failing school - all possibly the result of HIV infection, were found to be significantly associated with depression. Earlier identification of HIV infection in children with prompt initiation of ART could improve growth potential, immunity, physical appearance, and intellectual capacity and could therefore prevent many of these potential risk factors for depression amongst HIV-infected adolescents.

To our knowledge, our study is among the first to demonstrate a strong independent association between bullying and depression in sub-Saharan Africa; and the first to examine bullying related to HIV. Bullying is associated with severe mental health symptoms, including self-harm, and contributes independently to mental health problems [55]. In our study, 11.6 % of respondents reported being bullied for taking medications, and bullying victimization arose as the variable most significantly associated with depression in both the linear (beta coefficient 5.31 [95 % CI 3.19, 7.43]) and logistic (OR 4.20 [95 % CI 2.29]) regressions. Bullying has been established as an independent risk factor for depression in high-income countries and more recently is emerging as a risk factor in resource-limited settings [13, 56]. A recent study from India reported that children orphaned by AIDS were significantly more likely to report being bullied by friends or relatives than those orphaned due to other reasons [56]. As our study was cross-sectional, we cannot comment on whether or not children with depression are more likely to report bullying or if bullying leads to depression. Furthermore, our study did not directly measure potential HIV/AIDS-related stigma, although presumably bullying for taking medications is likely associated with stigma. However, a longitudinal study from South Africa reported that significant indirect effects via bullying victimization were obtained for both anxiety and depression scores. These effects were independent of poverty, HIV/AIDS-related stigma, and baseline mental health. The authors concluded that their data highlight bullying victimization as a potential target for future intervention efforts [57].

Due to the cross-sectional nature of this study, we cannot conclude any causal relationships. However, based on our data, we would also [57] recommend that bullying prevention programs may improve mental health outcomes for Malawian adolescents living with HIV and should be a focus of future research and programming. Some preliminary suggestions for interventions include teacher or school-based trainings to address bullying in HIV- and adolescent-support groups such as teen clubs, which may include curricula that directly address bullying. In our study, adolescents who were not in school or had completed only junior primary school had increased odds of depression as compared to adolescents with more education (OR 3.30 [1.54, 7.05]; *p* = 0.0051). Interventions that support children to remain in school may help to prevent depression. Stunting (low HAZ) and immunosuppression also were both independently associated with higher mean BDI-II scores. Interventions to facilitate prompt diagnosis of HIV and initiation of ART would prevent the on-towards effects of stunting and improve immunosuppression among HIV-infected adolescents.

The strengths of the study include the relatively large, geographically diverse sample and the measurement of an extensive range of variables previously shown or thought to be associated with depression among HIV-infected youth. In addition, we used both a validated depression-screening instrument (translated BDI-II) as a continuous outcome and a structured diagnostic interview (CDRS-R) as a categorical outcome. Finally, to our knowledge, this is the first study to examined factors associated with depression amongst HIV-infected adolescents in Malawi.

There were several limitations. As aforementioned, the design of the study was cross-sectional; therefore, we are unable to conclude any causal direction of the associations found and have no information regarding timing/onset of depression. Also although data collectors were trained on how to administer the SDBAQ, we did not perform inter-rater reliability testing. We also did not assess potential co-morbidities such as anxiety or intellectual disability. Due to depressive cognitive bias, youth with depression may have been more likely to report bullying/teasing. Finally, many of the variables were self-reported and, therefore, may be influenced by social desirability bias. Future studies could include longitudinal evaluations to measure temporal associations and should examine the relationship between depression and adherence in this group.

## Conclusion

In conclusion, we found that fewer years of schooling and bullying for taking medications were most clearly associated with depression, with the latter being the strongest independent determinant of depression. Programmes that help to identify and address the mental health needs of HIV-infected adolescents will contribute to solidifying global progress in expanding paediatric HIV treatment services.

## Abbreviations

ART: (Antiretroviral therapy)
BDI-II: (Beck depression inventory version-II)
CDRS-R: (Children’s depression rating scale-revised)
HAZ: (Height-for-age z-score)
PLWHA: (People living with HIV and AIDS)
SDBAQ: (Socio-demographic behavioural questionnaire)
